# Use of Genome-Wide Expression Data to Mine the “Gray Zone” of GWA Studies Leads to Novel Candidate Obesity Genes

**DOI:** 10.1371/journal.pgen.1000976

**Published:** 2010-06-03

**Authors:** Jussi Naukkarinen, Ida Surakka, Kirsi H. Pietiläinen, Aila Rissanen, Veikko Salomaa, Samuli Ripatti, Hannele Yki-Järvinen, Cornelia M. van Duijn, H.-Erich Wichmann, Jaakko Kaprio, Marja-Riitta Taskinen, Leena Peltonen, ENGAGE Consortium

**Affiliations:** 1 FIMM, Institute for Molecular Medicine Finland, University of Helsinki, Helsinki, Finland; 2 Public Health Genomics Unit, National Institute for Health and Welfare, Helsinki, Finland; 3 Department of Medical Genetics, University of Helsinki, Helsinki, Finland; 4 Obesity Research Unit, Department of Psychiatry, Helsinki University Central Hospital, Helsinki, Finland; 5 Finnish Twin Cohort Study, Department of Public Health, University of Helsinki, Helsinki, Finland; 6 Department of Chronic Disease Prevention, National Institute for Health and Welfare, Helsinki, Finland; 7 Department of Medicine, Division of Diabetes, University of Helsinki, Helsinki, Finland; 8 Department of Epidemiology and Biostatistics, Erasmus University Medical Center, Rotterdam, The Netherlands; 9 Institute of Epidemiology, Helmholtz Zentrum München, German Research Center for Environmental Health, Neuherberg, Germany; 10 Chair of Epidemiology, Institute of Medical Informatics, Biometry, and Epidemiology, Ludwig-Maximilians-Universität, Munich, Germany; 11 Klinikum Grosshadern, Munich, Germany; 12 National Institute for Health and Welfare, Helsinki, Finland; 13 Department of Medicine, Helsinki University Central Hospital, Helsinki, Finland; 14 Broad Institute, Massachusetts Institute of Technology, Cambridge, Massachusetts, United States of America; 15 The Wellcome Trust Sanger Institute, Cambridge, United Kingdom; Georgia Institute of Technology, United States of America

## Abstract

To get beyond the “low-hanging fruits” so far identified by genome-wide association (GWA) studies, new methods must be developed in order to discover the numerous remaining genes that estimates of heritability indicate should be contributing to complex human phenotypes, such as obesity. Here we describe a novel integrative method for complex disease gene identification utilizing both genome-wide transcript profiling of adipose tissue samples and consequent analysis of genome-wide association data generated in large SNP scans. We infer causality of genes with obesity by employing a unique set of monozygotic twin pairs discordant for BMI (n = 13 pairs, age 24–28 years, 15.4 kg mean weight difference) and contrast the transcript profiles with those from a larger sample of non-related adult individuals (N = 77). Using this approach, we were able to identify 27 genes with possibly causal roles in determining the degree of human adiposity. Testing for association of SNP variants in these 27 genes in the population samples of the large ENGAGE consortium (N = 21,000) revealed a significant deviation of *P*-values from the expected (*P* = 4×10^−4^). A total of 13 genes contained SNPs nominally associated with BMI. The top finding was blood coagulation factor *F13A1* identified as a novel obesity gene also replicated in a second GWA set of ∼2,000 individuals. This study presents a new approach to utilizing gene expression studies for informing choice of candidate genes for complex human phenotypes, such as obesity.

## Author Summary

Obesity has a strong genetic component and an estimated 45%–85% of the variation in adult relative weight is genetically determined. Many genes have recently been identified in genome-wide association studies. The individual effects of the identified genes, however, have been very modest, and their identification required very large sample sizes. New approaches are therefore needed to uncover further genetic variants that contribute to the development of obesity and related conditions. Much can be learned from studying the expression of genes in adipose tissue of obese and non-obese subjects, but it is very difficult to distinguish which genes' expression differences represent reactions to obesity from those related to causal processes. We studied monozygotic twin pairs discordant for obesity and contrasted the gene expression profiles of obese and lean co-twins (controlling for genetic variation) to those from unrelated individuals to try to discern the cause-and-effect relationships of the identified changes in gene expression in fat. Testing the identified genes in 21,000 individuals identified numerous new genes with possible roles in the development of obesity. Among the top findings was a gene involved in blood coagulation (Factor XIIIA1), possibly linking obesity with known complications including deep vein thrombosis, heart attack, and stroke.

## Introduction

In the current age of genome-wide association (GWA) studies using hundreds of thousands of single nucleotide polymorphisms (SNPs), obesity has been a popular phenotype to investigate. Obesity, often measured as the body mass index (BMI) and defined as BMI≥30 kg/m^2^, is important not only because of high and increasing prevalence, its strong association to many clinical complications such as type 2 diabetes, but also for the relative ease and availability of phenotypic measurement in most studies of human health. The heritability of BMI in twin and adoption studies range from 45%–85% [1] and still substantial, though somewhat lower in family studies. There are rare Mendelian syndromes with associated obesity and insights from rodent models and many linkage studies of obesity have identified chromosomal regions possibly harboring obesity genes on all chromosomes except the Y chromosome [2]. However, the known mutations account for only a small fraction of severe, early onset obesity and virtually none of the variability in adult overweight and obesity, while linkage studies and candidate gene studies have been plagued by a lack of replication [2]. Even meta-analyses of linkage have failed to reveal new genes.

The advent of GWA studies of BMI has lead to novel, robust findings. However, the genes identified so far have been able to explain only a small fraction of the variation in BMI; *FTO*, the first gene unequivocally associated with BMI was estimated to explain ∼1% of the total variance in the discovery sample set [3]. That study involved the genotyping of more than 30,000 individuals and the identification of the next most convincing, common BMI associated gene *MC4R* required the combined analysis of more than 77,000 individuals [4]. The most recent results from the GIANT Consortium with more than 91,000 analyzed DNA samples were able to identify an additional six new loci for BMI, together explaining 0.40% of the total variance in BMI [5]. Significantly, these six new loci in conjunction with the known associations at *FTO* and *MC4R* only accounted for 0.84% of the total variance [5], an observation that highlights the “winner's curse” phenomenon whereby effect sizes of associated variants are often exaggerated in the initial study relative to follow-up studies [6]. A simultaneous publication reported similar findings in a study consisting largely of the Icelandic DeCode data set. There, four loci identified in the GIANT consortium study and seven new loci influencing BMI were uncovered (each with explanatory power comparable to the variants from the GIANT consortium study) [7].

Even if only a fraction of the observed heritability of BMI consists of genes with universal effects in most, if not all populations, the remaining obesity predisposing genes surely number in the hundreds, if not thousands. However, their individual contributions are expected to be increasingly more modest and as such, more difficult to identify by (now) conventional means of GWA. The uncovering of these remaining genes will either call for unrealistically large sample sizes, or rather, novel methods for nominating potential candidate genes likely to be causally involved with the trait of interest.

The integration of genome-wide expression data from relevant tissues with SNP scans is a promising approach for identifying novel genes involved in the development of complex disease traits such as obesity. Microarray data and SNP scans from individuals have been combined in order to map variants (eQTLs) that control the expression of nearby genes in segregating populations [8], [9]. While these studies have identified large numbers of *cis*-acting variants and a smaller number of *trans*-acting variants, they are not powered to link the changes in gene expression with the development of complex diseases and their trait components.

**Figure 1:**
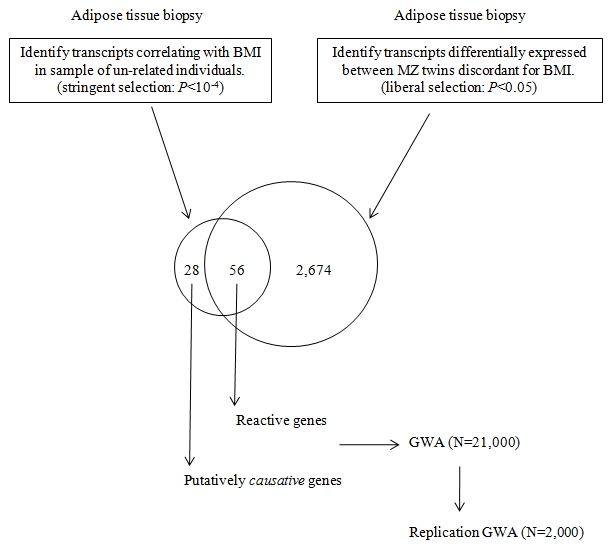
Diagram of the study design. Adipose tissue biopsies from MZ twins discordant for BMI and from a collection of unrelated individuals are analyzed by expression array. Contrasting the differences in transcript profiles allows for the identification of putatively causative genes effecting BMI. Subsequent GWA testing of SNP variants in the identified genes allows for evaluation of genetic variation that may predispose to obesity.

Correlation of gene expression with a phenotype such as BMI however does not imply causation and the difficulty comes in discerning the transcripts that merely react to the disease state in target tissues from those that are actually related to causal processes. This is an especially challenging task in human studies, where the system cannot be perturbed in the same sense as is possible with animal models or cell lines. In order to circumvent some of these challenges and to infer causality, we studied adipose tissue gene expression in a unique collection of young monozygotic twin pairs without other co-morbidities discordant for BMI [n = 13 pairs, age 24–28 years, 15.4 kg mean weight difference, with no significant height differences (<3 cm)] as well as in a set of 77 unrelated individuals in order to establish a set of genes most likely to be related to causal processes in controlling the degree of human adiposity. Given that monozygotic twins are identical at the level of DNA sequence (precluding somatic mutations), differences in the expression of the genes encoded therein are the result of regulatory and/or epigenetic changes in response to lifestyle and the environment. By this logic, the genes with significant expression differences in the adipose tissue between the lean and obese monozygotic co-twins in discordant pairs can be designated as *reactive* to the obesity. In contrast, genes whose transcript levels in adipose tissue correlate significantly with BMI in a large sample of non-related individuals will be a mixture of reactive genes as well as genes related to *causal* processes. While it would be expected that there would be a significant degree of overlap in the list of genes identified in the two different sample sets, the subset of genes that correlate significantly with BMI *only* in the non-related sample would present as candidates for primarily being related to causal processes. Genes identified as possibly “causal” to obesity can subsequently be interrogated for association with BMI in a large GWA study cohort in order to establish whether the genes harbor sequence variants that effect their expression in *cis*. This study design is presented in Figure 1.

## Results

### MZ twin pairs discordant for BMI

The precise effects of acquired obesity have been difficult to investigate due to the complex interaction of genes and the environment that are thought to be involved in its development. The correlation of BMI as measured between pairs of genetically identical twins representative of the population is very high, with a correlation coefficient of 0.79 in the FinnTwin16 study from which the twins here analyzed were recruited [10]. It is the rare cases of significantly discordant twin pairs that allows for the most detailed analysis of the environmental effects, in this case of acquired obesity. With the MZ co-twin control design applied here, we were able to control for gender, age, cohort effects as well as other exposures and experiences of childhood that are shared by siblings in a family. Thus, we carefully assess the effects that obesity has on the transcription of genes in adipose tissue.

A thorough description of these 13 pairs of MZ twins (age 24–28 years, eight male pairs and six female pairs) most discordant for BMI has been published earlier by our group [11]. Briefly, the selected pairs represent 5% extreme ends in divergence of phenotypes and the obese co-twins were on average 15.4 kg (22%) heavier than the lean co-twin. They exhibited a significantly larger fat mass in every depot measured, including subcutaneous, intra-abdominal and liver fat (*P*<0.002). Consequently the obese twins were also much more insulin resistant, as evidenced by the lower M-value and higher fasting serum insulin levels. Obesity in the heavier co-twins had developed after post-pubertal adolescence [12] and thus the metabolic abnormalities represent consequences of early stages of excess adiposity (Table S1).

### Sample of unrelated individuals

The sample of unrelated individuals used consisted of 77 individuals from the same Finnish population as the MZ twins. Subjects had been recruited as a part of a large study on familial dyslipidemias and consisted of 41 women and 36 men. Phenotypic measures (±1S.D.) were as follows: BMI 26.6±4.1 kg/m^2^ (range 20.1–37.8 kg/m^2^), age at adipose tissue biopsy 51±11.6 years (range 27.4–68.9 years). They thus exhibit a wide and representative range of BMI.

### Gene expression changes related to increased adiposity

First, in order to establish the list of genes most likely to be “reactive” to the obese state, samples of subcutaneous adipose tissue from the discordant MZ twins were analyzed. Following co-twin normalization of the expression data, we identified 2,674 transcripts differentially regulated in the adipose tissue of the obese twins compared to that of the lean co-twin (non-parametric Welch t-test, using a liberal test value, i.e. *P*<0.05).

Next, in a data set consisting of adipose tissue samples from 77 unrelated individuals, using stringent criteria (Pearson correlation *P*<10^−4^) we identified 84 transcripts that correlated with BMI. Of these, 56 (or 2/3) were shared with those identified in MZ study samples and designated as “reactive”. The remaining 28 transcripts encoded by 27 unique genes that, while strongly correlated with BMI, were not differentially regulated in the discordant MZ twins and as such were candidates for being related to causal processes (Table 1). The *P*-value for the Pearson correlation between BMI and gene expression for these genes in the unrelated sample ranged from 2.5×10^−6^ for *HADHA*, to 9.99×10^−5^ for *SAP30BP*. By definition, these genes were not differentially expressed between the obese and lean co-twins (*P*-values 0.35 and 0.56 respectively). The *P*-values for correlation of expression level with BMI among the twins ranged from 0.051 to 0.849. A number of the known obesity associated genes were not included in the analyses, as they were filtered out either as reactive (Leptin), showed no significant correlation with BMI in either sample (*FTO*), or were not expressed in fat (*MC4R*).

**Table 1:** Relationship between adipose tissue gene expression and BMI for the putative causative genes among 77 unrelated subjects (Pearsonian correlation and associated *P*-value), fold change, and *P*-value for the intrapair difference in gene expression between obese and lean members of obesity discordant MZ pairs.

		Unrelated subjects		Discordant MZ twin pairs	
Gene symbol	Affymetrix probe-ID	Pearson Correlation	*P*-value^a^	Obese/Lean Expression^b^	*P*-value^c^
*SAP30BP*	217965_s_at	−0.51	9.99×10^−5^	1.00	0.56
*MRPL9*	209609_s_at	−0.43	9.57×10^−5^	0.89	0.72
*C20orf198*	226149_at	−0.35	9.26×10^−5^	0.93	0.45
*IVNS1ABP*	206245_s_at	0.46	8.86×10^−5^	1.24	0.08
*SPSB3*	46256_at	−0.52	8.32×10^−5^	0.93	0.06
*SART1*	200051_at	−0.34	8.29×10^−5^	1.01	0.83
*ST3GAL6*	210942_s_at	−0.52	8.27×10^−5^	0.77	0.05
*FAHD2A*	222056_s_at	−0.41	8.05×10^−5^	0.97	0.53
*mRNA AK125162*	231040_at	−0.42	7.95×10^−5^	0.83	0.85
*SH3BGR*	204979_s_at	0.32	5.0×10^−5^	1.13	0.11
*NPY1R*	205440_s_at	0.42	5.0×10^−5^	1.15	0.14
*EIF2B4*	209429_x_at	−0.48	4.83×10^−5^	1.07	0.62
*EIF4EBP1*	221539_at	−0.54	4.55×10^−5^	0.82	0.08
*AATF*	209165_at	−0.46	4.26×10^−5^	1.03	0.44
*PRDX6*	200844_s_at	−0.48	3.39×10^−5^	0.93	0.18
*SH3BGRL*	201312_s_at	0.38	3.33×10^−5^	1.12	0.18
*PCBD1*	203557_s_at	−0.56	3.1×10^−5^	0.88	0.05
*PRSS23*	226279_at	0.33	2.78×10^−5^	1.11	0.07
*BC007882*	225657_at	−0.28	2.72×10^−5^	0.96	0.67
*ST3GAL6*	213355_at	−0.47	2.48×10^−5^	0.97	0.24
*TMEM101*	225004_at	−0.36	1.79×10^−5^	0.96	0.11
*F13A1*	203305_at	0.41	9.59×10^−6^	1.13	0.11
*TRAP1*	201391_at	−0.38	6.53×10^−6^	0.98	0.43
*CRY2*	212695_at	−0.41	5.35×10^−6^	0.92	0.22
*PPIE*	210502_s_at	−0.6	4.9×10^−6^	0.97	0.54
*C16orf62*	225772_s_at	−0.43	4.9×10^−6^	0.83	0.06
*RNASE4*	213397_x_at	−0.44	2.68×10^−6^	0.86	0.09
*HADHA*	208630_at	−0.5	2.5×10^−6^	1.03	0.35

Table has been sorted according to the *P*-value of the Pearson correlation.

a *P*-value for the Pearson correlation between BMI and gene expression in the set of 77 unrelated individuals.

b Median fold difference in gene expression between the obese and lean co-twins. Values greater than 1.00 represent a higher expression level in the obese twin.

c *P*-value for non-parametric Welch t-test of gene expression between lean and obese co-twins of obesity discordant pairs.

### Mining the ENGAGE consortium GWA data

**Figure 2:**
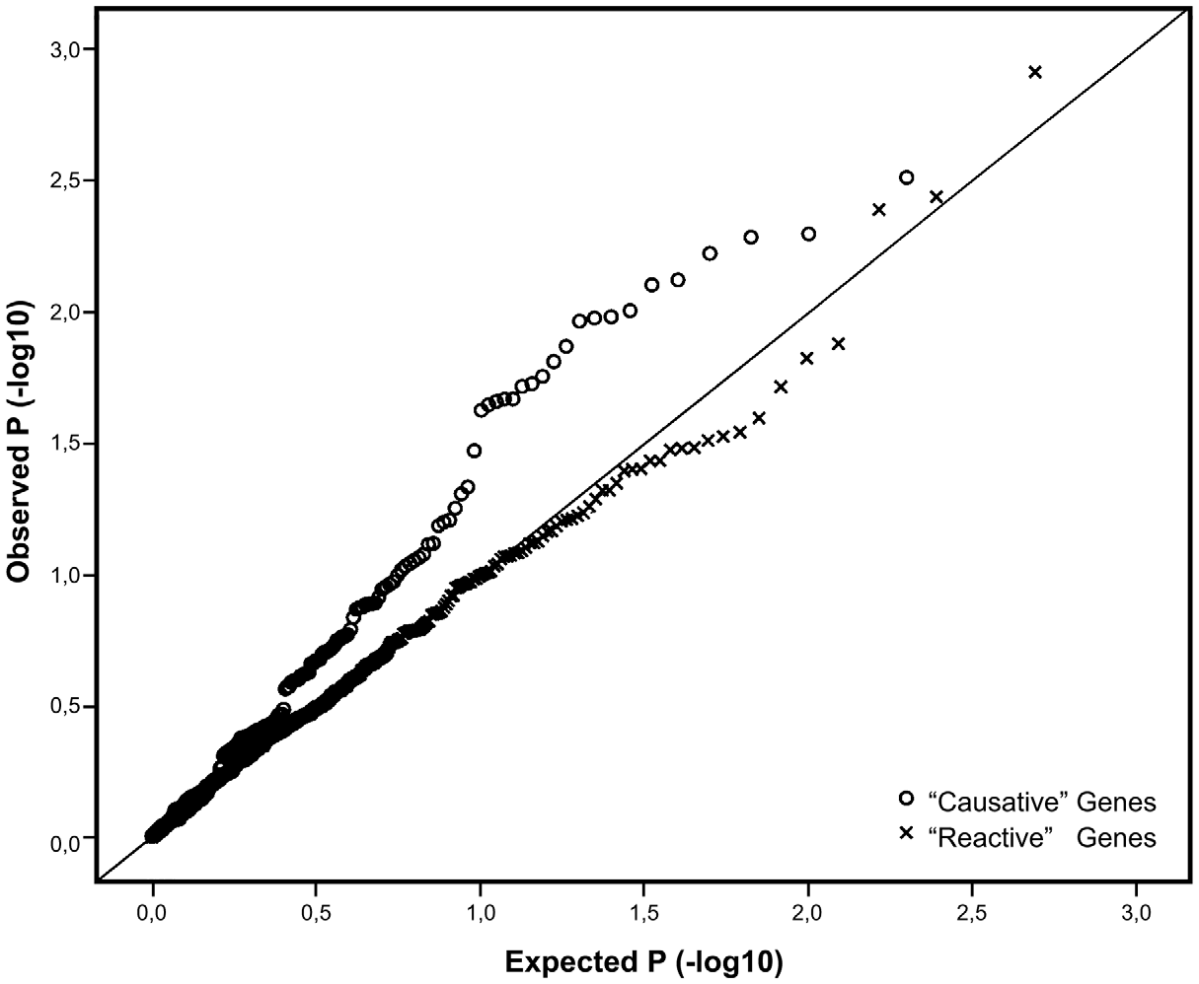
Q-Q plot comparing the distribution of *P*-values for the causative and reactive gene sets. SNP variants in the reactive genes are marked by “x” while SNP variants in the putatively causative genes are marked with open circles. Line y = x represents the expected distribution of *P*-values, following the uniform distribution.

In order to assess, whether any of the genes identified as putatively causative in influencing obesity also harbored SNP variants associated with the trait, we employed the large ENGAGE consortium study sample consisting of more than 21,000 GWA scans from well characterized European population cohorts, not ascertained for any specific trait. An analysis of the 197 SNPs, -representing the 27 putatively “causative” genes revealed a significant excess of small *P*-values, differing from the expected uniform distribution (*X* ^2^ *P*-value 4×10^−4^). By contrast, the 498 SNPs representing the 48 genes “reactive” to obesity did no better than expected by chance (*X* ^2^ *P*-value 0.59), as witnessed by the Q-Q plots with very different profiles for the two gene sets (Figure 2). It is possible for such Q-Q plots to be inflated due to a large number of associating SNPs in one or a few genes (with high local LD), but re-drawing these plots while retaining only the best single SNP per gene did not significantly change their profiles (Figure S1).

Out of the 27 tested putatively causative genes, 13 genes harbored a total of 23 SNPs that were nominally associated with BMI in our sample (*P*<0.05) (Table 2). The top hit was SNP rs2274393 (*P* = 0.003) located in an intron of the 177 kb gene encoding the A1 subunit of the coagulation factor XIII (*F13A1*), a gene previously associated with the risk of venous thromboembolism [13]. Altogether 7 SNPs in the *F13A1* gene associated with BMI in the 21,000 ENGAGE samples (Figure S2). Among the other genes for which positive evidence of association (*P*<0.05) could be shown were the sialyltransferase *ST3GAL6*, ribonuclease 4 (*RNASE4*), peroxiredoxin 6 (*PRDX6*), SH3 domain binding glutamic acid-rich protein (*SH3BGR*), the influenza virus NS1A binding protein (*IVNS2ABP*), the apoptosis antagonizing transcription factor (*AATF*), transmembrane protein 101 (*TMEM101*) as well as some novel transcripts with no known functions.

**Table 2:** SNPs associating with BMI among the putatively causative genes in the ENGAGE consortium GWA sample.

Gene name	MAF	SNP	N	P-value women	P-value men	Overall P-value
F13A1	0.252	rs2274393	20,812	0.0084	0.1413	0.0030
ST3GAL6	0.463	rs865474	20,789	0.0571	0.0350	0.0050
F13A1	0.433	rs406238	20,782	0.0077	0.2417	0.0051
ST3GAL6	0.294	rs7628381	20,795	0.0266	0.1016	0.0059
RNASE4	0.269	rs3094	20,718	0.0021	0.6363	0.0074
C20orf198	0.156	rs1739652	4,584	0.0599	0.0599	0.0078
F13A1	0.152	rs11243069	20,805	0.0095	0.3712	0.0097
F13A1	0.220	rs9504743	20,806	0.0991	0.0414	0.0103
C16orf62	0.337	rs3782323	20,812	0.0616	0.0782	0.0104
PRDX8	0.262	rs6675929	20,797	0.0287	0.1753	0.0107
SH3BGR	0.234	rs2837035	20,806	0.0338	0.1876	0.0133
IVNS1ABP	0.433	rs1889976	20,807	0.0825	0.0876	0.0152
SH3BGRL	0.261	rs5959087	20,814	0.0741	0.1159	0.0173
SH3BGRL	0.262	rs12389790	20,777	0.0816	0.1122	0.0185
SPSB3	0.176	rs17559	16,029	0.0022	0.9365	0.0189
F13A1	0.266	rs9328347	20,808	0.1760	0.0474	0.0211
F13A1	0.347	rs387528	4,572	0.1031	0.1031	0.0212
F13A1	0.185	rs3024443	20,813	0.2437	0.0271	0.0216
AATF	0.214	rs11871099	20,803	0.1250	0.0825	0.0223
AATF	0.342	rs1564796	20,781	0.1268	0.0869	0.0233
IVNS1ABP	0.431	rs10911707	4,574	0.1323	0.1323	0.0333
TMEM101	0.432	rs1618809	20,778	0.1937	0.1183	0.0458
EIF4EBP1	0.223	rs6605631	4,584	0.1633	0.1633	0.0486

Table has been sorted according to the overall *P*-value.

MAF: minor allele frequency.

While the potential “reactive” genes did not deviate significantly from the expected uniform distribution, the top 4 SNPs were of some interest, as they were all located in the *URB* gene (*u*p*r*egulated in *b*ombesin receptor subtype 3 knockout mouse), recently implicated as having a role in human obesity [14]. The best *P*-value was obtained for intronic SNP rs9870432 (*P* = 0.001) (Table 3). The association signal for URB was coming solely from the women in the analysis, suggesting a possible gender-specific role for this gene in influencing obesity and BMI.

**Table 3:** SNPs associating with BMI among the reactive genes in the ENGAGE consortium GWA sample.

Gene name	MAF	SNP	N	P-value women	P-value men	Overall P-value
*URB*	0.457	rs9870432	20,574	0.0008	0.2973	0.0012
*URB*	0.314	rs6807798	20,659	0.0010	0.5546	0.0036
*URB*	0.384	rs10511316	20,644	0.0015	0.5113	0.0040
*URB*	0.372	rs2279531	20,613	0.0095	0.4464	0.0130
*FLJ45244*	0.148	rs3814816	20,437	0.1010	0.0631	0.0147
*C12orf30*	0.160	rs7297415	20,657	0.0856	0.1071	0.0189
*PALLD*	0.467	rs7670597	20,654	0.6463	0.0028	0.0249
*CIDEA*	0.376	rs6505744	20,658	0.1039	0.1397	0.0282
*DAPK2*	0.024	rs2034099	4,427	0.0019	0.8398	0.0293
*MRC1*	0.089	rs537085	4,407	0.2544	0.0531	0.0304
*ABCC1*	0.431	rs3887893	20,657	0.0749	0.2300	0.0323
*ADAM12*	0.288	rs1459709	20,665	0.1430	0.1125	0.0326
*CCL2*	0.322	rs991804	20,666	0.1117	0.1547	0.0329
*ADAM12*	0.493	rs1278377	20,654	0.0127	0.7924	0.0363
*CLMN*	0.375	rs12894609	20,650	0.0696	0.2778	0.0364
*PALLD*	0.470	rs6852874	20,311	0.1212	0.1719	0.0390
*CCL2*	0.388	rs4795893	20,661	0.0201	0.6731	0.0391
*ABCC1*	0.131	rs16967126	20,653	0.6589	0.0058	0.0397
*ABCC1*	0.167	rs152029	20,658	0.0362	0.5482	0.0443
*PALLD*	0.429	rs17542430	20,623	0.4509	0.0274	0.0471
*CPVL*	0.194	rs245857	20,587	0.3262	0.0525	0.0472

Table has been sorted according to the overall *P*-value.

MAF: minor allele frequency.

### *F13A1* association with BMI replicates in GenMets study

In order to assess whether any of the associations observed in the large ENGAGE sample were replicable, we utilized a smaller available GWA study sample, namely the GenMets study - a case/control study with ∼2,000 individuals designed to investigate the genetics of metabolic syndrome. The top-hit among the putatively causative genes was again a SNP in the *F13A1* gene (rs714408 *P* = 0.002) (Table S2). A meta-analysis of the two combined study samples (N∼23,000) increased the significance of this *F13A1* SNP to *P* = 0.0004). In this replication dataset the effect size of this rs714408 variant on BMI was −0.2519 (±0.1472) BMI units per G allele carried. This equates to 0.14% of the variance in BMI. No other genes implicated in the ENGAGE study replicated in this smaller GenMets study sample, but the Q-Q plots bore the same distinct differences as in Figure 2 with the putatively causal genes outperforming the reactive genes (Figure S3). Among the reactive genes, the *URB* variants associated with BMI in the ENGAGE sample did not replicate in the GenMets study (best *P* = 0.46).

## Discussion

While BMI is a highly heritable and much investigated trait in humans, the success in identifying common predisposing genetic variants has been relatively modest with until very recently, only two solidly established gene findings to date, namely the *FTO* [3] and *MC4R* [4] genes, each explaining only a small fraction of the variance. The newest study identifying 6 additional obesity predisposing variants in combination with *FTO* and *MC4R* explained only 0.84% of the variance in BMI. While highlighting the complex genetic and environmental background of obesity, this realization also calls for novel designs in order to identify the remaining genes, each arguably with increasingly more modest effect sizes, or having characteristics that are not captured by the current generation of SNP-chips. Utilizing a unique experiment of nature, namely monozygotic twin pairs discordant for obesity and contrasting the obese/lean co-twin differences in expression profiles in adipose tissue with those from a collection of unrelated individuals, we were able to circumvent some of the difficulties experienced by other studies and infer putative causality of the observed expression changes in relation to obesity. Testing for association in a large collection of GWA data from European population cohorts (N = 21,000) we were able to illustrate the power of this data mining scheme in uncovering novel genetic variants associated with complex human traits like BMI.

In addition to genetic variation, the expression levels of genes, as measured by appropriate microarrays, can be affected by several factors including environmental variation, epigenetic modifications as well as by experimental conditions of *in vitro* cell lines. Using monozygotic twin pairs discordant for obesity in our experiment allowed for controlling for many of these factors, like gender, age, early developmental environment as well as cohort-effects. Being able to designate the differentially expressed transcripts as reactive to the obesity allowed for the identification in the unrelated sample set of transcripts with a putatively causative role in effecting human obesity. SNP variants in the genes designated as “causative” based on the MZ data performed considerably better in the test for association with BMI than the variants in the reactive genes, as evident in Figure 2.

A total of seven BMI associated SNP variants, including the top SNP of the study were located in the *F13A1* gene. Multiple *F13A1* SNPs were also associated with BMI in the replication GWA cohort, with the strongest evidence for association in the final meta-analysis for SNP rs714408 (*P* = 0.0004). In our data set, *F13A1* transcript levels were also significantly associated with BMI (Pearson correlation 0.41, *P*-value 9.6×10^−6^) among the samples from unrelated individuals. F13A1, coded for by a 177 kb gene in chromosome 6p25.1 is a subunit of coagulation factor XIII, the last zymogen to become activated in the blood coagulation cascade and responsible for forming the crosslinks between fibrin molecules that stabilize a clot [15]. A common V34L (rs5985) variant in *F13A1* has previously been associated with venous thromboembolism, and supported by a recent large meta-analysis [13]. The V34L variant was genotyped in this study, but failed to associate with BMI directly in the ENGAGE set (*P* = 0.70). In light of this, it does not seem like the V34L polymorphisms is the one underlying the BMI association observed here, neither do we believe to have captured the functional *F13A1* variant itself. The linkage disequilibrium between the V34L polymorphism and the rs714408 variant here identified is R^2^ = 0.142. The polymorphism(s) may influence F13A1 in several ways by either modulating its expression in the cells, by effecting its stability, or by changing its activity in the circulation, as has been shown to be the mode of action of the V34L polymorphism [16]. Further genotyping, sequencing and functional analyses are required to elucidate the functionality of the polymorphisms here identified. Aside from a report describing a correlation of *F13A1* expression with liver fat in humans [17], this is to our knowledge the first instance where *F13A1* has been implicated in obesity. Given that obesity is well known to predispose to the development of deep vein thromboses and it is considered a chronic prothrombic state [16], these observations may open new avenues for studying the link between obesity and thrombosis. This may also help account for the increased risk of cardiovascular disease in obese subjects.

Circumstantial evidence from a number of other studies lends support for the possible role in obesity of several other genes identified in the present study. The second best association with BMI was for SNP rs865474 located in the gene *ST3GAL6*, coding for a sialyltransferase. While no known link with obesity exists for this gene, a previous study found its expression to be highly variable and much more highly correlated between related individuals in lymphoblastoid cell lines, suggesting that the expression of this gene is under strong genetic control [18]. Our results in the MZ twins agree with this finding and given the strong correlation of the transcript with obesity in the unrelated individuals (Pearson correlation −0.47, *P* = 2.48×10^−5^), this makes *ST3GAL6* a potentially interesting new candidate. The third associating gene *RNASE4* is a ribonuclease previously been shown to be upregulated in adipose tissue of women following a low calorie diet [19] and to be markedly increased after a 24 hour treatment with cortisol [20]. In mice *RNASE4* has been shown to be regulated in the liver by dietary fat [21]. *PRDX6*, an antioxidant enzyme with Ca-independent phospholipase A2 activity [22], was identified in a recent study as a possible candidate gene underlying a novel obesity locus on chromosome 1q24 in an isolated population of Cilento [23]. The expression of *SH3BGR* was previously reported to be downregulated in adipose tissue following weight reduction [24], a finding consistent with the positive correlation of transcript levels with BMI identified here (Pearson correlation 0.32, *P* = 5.0×10^−5^). An interesting candidate for obesity is *IVNS1ABP* (influenza virus NS1A binding protein). While it has no known function with a relation to obesity, a recent study of Oceanian population genetics utilizing genome-wide SNP analyses identified the *IVNS1ABP* gene as having undergone a selective sweep in the Oceanian population [25]. Among other such genes identified in the study were the VLDL-receptor and other lipid metabolism related genes, suggesting that *IVNS1ABP* may represent just the kind of obesity predisposing thrifty genes that have been hypothesized to be enriched in the Oceanian populations with a well known current epidemic of obesity and type 2 diabetes in conjunction with the westernization of their diets and lifestyle. Another very plausible candidate identified is the neuropeptide Y receptor 1 (*NPY1R*), a receptor for a neuropeptide exhibiting a diverse range of physiologic activities including effects on food intake [26].

While a whole number of genes had been filtered out from the analyses as being clearly reacting to the obesity, there is no reason to believe that they too could not contain variants that effect the development of obesity. Genes that are designated as “reactive”, but still associate with obesity may be ones whose transcript levels are affected by the developing obesity but that concurrently harbour variants predisposing an individual to weight gain. In fact, the top result (top 4 SNPs) among the genes identified as reactive, was *URB*, a gene linked with obesity in mice [27] and recently implicated as having a role in obesity in humans [14]. Our results here support a gender specific role for SNP variants in *URB* in the development of obesity. A few of the transcripts designated as “causative” also approach nominal significance in regards to correlation with BMI (Table 1), supporting the notion that some genes could eventually be classified as both “causative” and “reactive”.

The power of this study lies in the availability of the obesity discordant monozygotic twins that enable the dissection of cause and effect relationships between gene expression and obesity. A similar approach could be utilized for many other complex traits where discordant MZ twins and relevant tissue biopsies are available, for example muscle biopsies from pairs discordant for physical activity. Such collections of twins are admittedly rare, but other approaches (animal clones exposed to different environments, human subjects that have gained or lost significant amounts of weight) can approximate the experimental set up applied here.

The most recent GWA studies on obesity give considerable hope that additional variants, each explaining in the range of 0.1% of the variance will be identified in new, large scale GWA studies utilizing tens of thousands of samples [5], [7]. However, novel integrative methods as described here are called for in order to uncover the remaining variants. Such alternative designs, combined with comprehensive sequencing of candidate genes and loci, should give us a clearer picture of the allelic architecture and help to discern the relative contributions of rare and common variants to human obesity.

## Materials and Methods

### Study participants for the adipose tissue biopsies

Adipose tissue biopsies from two study samples originating from the Finnish population were used. Both studies were performed according to the principles of the Helsinki Declaration.

The twins from discordant pairs were recruited from a population-based longitudinal study of five consecutive birth cohorts (1975–1979) of twins, their siblings and parents (n = 2,453 families), identified through the national population registry of Finland [28]. The twins selected for this study represented the top 5% most obesity-discordant MZ twin pairs (one co-twin not obese [BMI∼25 kg/m^2^], and the other one obese [BMI∼30 kg/m^2^]), with no significant height differences (<3 cm) or chronic illness. Of the 18 pairs thus identified, 13 pairs participated in the adipose tissue biopsy. The study subjects have been previously thoroughly described [11]. The protocol was approved by the Ethical Committee of the Helsinki University Central Hospital.

The unrelated study subjects were recruited as a part of the European Multicenter Study on Familial Dyslipidemias in patients with Premature Coronary Heart Disease (EUFAM) and consisted of both healthy and affected individuals. The collection of subjects has been described earlier [29], [30]. The Ethical Committee of the Department of Medicine, Helsinki University Central Hospital approved the study.

All fat biopsies were collected under local anesthetic by a needle aspiration of periumbilical subcutaneous fat, immediately frozen in liquid nitrogen and stored at −80°C for later extraction of total RNA using the RNeasy Lipid Mini Kit (Qiagen) according to manufacturer's instructions. Quality of RNA was analyzed using the 2100 Bioanalyzer platform (Agilent Technologies).

### Gene expression arrays

Two micrograms of total RNA were treated according to conventional Affymetrix eukaryotic RNA labeling protocols (Affymetrix, Santa Clara, CA). Fifteen micrograms of biotin labeled cRNA was fragmented according to Affymetrix eukaryotic sample protocol. Hybridization, staining and washing of the Affymetrix U133 Plus 2.0 chips were performed using the Affymetrics Fluidics Station 450 and Hybridization oven 640 under standard conditions. Prior to analysis, raw expression data were normalized using the GCRMA algorithm, separately for the MZ twin and non-related individuals. Analysis of the adipose tissue expression data was done using the GeneSpring GX 7.3 software (Agilent Technologies).

### Identifying the “reactive” versus “causative” transcripts

In order to include in the expression analyses only those probes with reliably high signal, the expression data was first filtered according to expression level so that only probes with expression value≥50 in at least ½ of the samples were included. Of the ∼54,000 probesets available on the array 17,026 (∼31%) passed the filtering and were considered for analysis. Given the unique set-up involving MZ twins, following standard GC-RMA normalization of the expression signals, we next performed a “co-twin normalization” procedure which involved dividing the obese twin's expression values with those of the non-obese co-twin in order to correct for the identical genetic background (and by definition also for gender and age). Using a liberal threshold for significance (non-parametric Welch t-test *P*<0.05) we identified 2,674 transcripts differentially regulated in the adipose tissue of the obese twins.

Next, in a data set consisting of adipose tissue samples from 77 unrelated individuals, using stringent criteria (Pearson correlation *P*<10^−4^) we identified 84 BMI correlated transcripts. Of these, 56 were shared with those identified in MZ study samples and designated as “reactive”. The remaining 28 transcripts encoded 27 unique genes that, while strongly correlated with BMI, were not differentially regulated in the discordant MZ twins and as such were candidates for being related to causal processes.

For the twin samples a liberal threshold was chosen in order to avoid unintentionally designating them as “causative”. This, contrasting with the stringent criteria for significance applied for the sample of unrelated individuals was done in order to avoid false-positives in the last stage of filtering.

### Meta-analysis of GWA data

For the genes identified through the expression profiling as being either “causative” or “reactive”, we retrieved all the genotyped SNPs located in the coding sequence positions ±1 kb, as annotated in the UCSC genome browser (March 2006 build). Altogether 197 SNPs in these genes were tested for association with BMI in the large set of ENGAGE cross-sectional population cohorts from populations of European origin (N = 21,000). All available SNPs were similarly retrieved for the 49 genes (represented by 56 Affymetrix probes) that were designated as “reactive”. One of the genes was X-chromosomal for which no genotypes were available. This resulting set of 48 genes was used as the control gene set against which we compared the association findings with the genes designated putatively causative. Within each of the 16 ENGAGE cohorts logarithmized BMI measures were stratified by sex and in each of the stratum outcomes were adjusted for age using linear regression model. The residuals resulting from these models were then standardized and used as outcome measures in the association analyses. Association analyses were performed with linear regression assuming additive mode of inheritance. These cohort specific results were combined into a fixed effects meta-analysis with reciprocal weighting on the square of standard errors of the effect size estimates.

### Replication GWA study sample: GenMets

As a replication study sample we utilized the GenMets study collected from the Finnish population for an investigation of the genetics of metabolic syndrome. GenMets individuals were sampled from a Finnish population-based Health 2000 study. Details of the Health 2000 study and phenotype measurements have been reported earlier [31]. The sample consisted of 919 individuals with non-diabetic metabolic syndrome, and 932 individuals without the metabolic syndrome, as defined by the International Diabetes Federation [32]. The individuals were genotyped using Illumina HumanHap 610 K chip and imputed using the Hapmap 1+2 CEU reference data.

### Statistical analysis

GCRMA normalized gene expression values in the sample of unrelated individuals were evaluated for correlation with BMI using the Pearson correlation as implemented in SPSS 11.0 for Windows (SPSS Inc., Chicago, IL, USA). A *P*-value of <10^−4^ was considered significant. Following co-twin normalization, differential expression of transcripts between the BMI discordant MZ twin pairs was evaluated in Gene Spring GX 7.3 software (Agilent Technologies) by the non-parametric Welch t-test with *P*<0.05 considered significant for further analysis. The Pearson *X* ^2^ test of categorized *P*-values was used to test for deviation of the distribution of observed *P*-values from the uniform distribution. In GenMets GWA analyses logarithmized BMI measures were adjusted for sex, age and metabolic syndrome affection status using linear regression model Metabolic syndrome status was used as a covariate in the linear model to minimize the effect of the study design. The main component behind the metabolic syndrome definition used in GenMets is waist circumference, although correlated with BMI, it may have a different genetic background from BMI. However, by adjusting we may run into over-adjusting and losing some statistical power. The residuals resulting from these models were standardized and used as outcomes as in ENGAGE analyses, Association analysis was conducted using PLINK [33] option linear.

## Supporting Information

Figure S1 Q-Q plot for causative and reactive genes (only best SNP per gene).
(0.06 MB TIF)

Figure S2 Association signals in *F13A1*. The upper panel displays the genotyped SNPs and their −log_10_ p-values. The panel below this displays the LD structure with a black line indicating the cM/Mb rate. High peaks correspond to recombination hotspots (with low LD). The red line indicates the genetic distance from the SNP with the lowest p-value. In the bottom most panel the red bar marks the location of the *F13A1* gene.
(2.71 MB TIF)

Figure S3 Q-Q plot of reactive and causative genes in the GenMets study.
(0.07 MB TIF)

Table S1 Physical and biochemical characteristics of the obesity-discordant monozygotic twin pairs (n = 13).
(0.04 MB DOC)

Table S2 SNPs associating (at *P*<0.05) with BMI among the putatively causative genes in the GenMets study, ordered by overall *P*-value.
(0.04 MB DOC)

## References

[1] Schousboe K, Willemsen G, Kyvik KO, Mortensen J, Boomsma DI (2003). Sex differences in heritability of BMI: a comparative study of results from twin studies in eight countries.. Twin Res.

[2] Rankinen T, Zuberi A, Chagnon YC, Weisnagel SJ, Argyropoulos G (2006). The human obesity gene map: the 2005 update.. Obesity (Silver Spring).

[3] Frayling TM, Timpson NJ, Weedon MN, Zeggini E, Freathy RM (2007). A common variant in the FTO gene is associated with body mass index and predisposes to childhood and adult obesity.. Science.

[4] Loos RJ, Lindgren CM, Li S, Wheeler E, Zhao JH (2008). Common variants near MC4R are associated with fat mass, weight and risk of obesity.. Nat Genet.

[5] Willer CJ, Speliotes EK, Loos RJ, Li S, Lindgren CM (2009). Six new loci associated with body mass index highlight a neuronal influence on body weight regulation.. Nat Genet.

[6] Kraft P (2008). Curses—winner's and otherwise—in genetic epidemiology.. Epidemiology.

[7] Thorleifsson G, Walters GB, Gudbjartsson DF, Steinthorsdottir V, Sulem P (2009). Genome-wide association yields new sequence variants at seven loci that associate with measures of obesity.. Nat Genet.

[8] Gilad Y, Rifkin SA, Pritchard JK (2008). Revealing the architecture of gene regulation: the promise of eQTL studies.. Trends Genet.

[9] Dermitzakis ET (2008). From gene expression to disease risk.. Nat Genet.

[10] Kaprio J (2006). Twin studies in Finland 2006.. Twin Res Hum Genet.

[11] Pietilainen KH, Naukkarinen J, Rissanen A, Saharinen J, Ellonen P (2008). Global transcript profiles of fat in monozygotic twins discordant for BMI: pathways behind acquired obesity.. PLoS Med.

[12] Pietilainen KH, Rissanen A, Laamanen M, Lindholm AK, Markkula H (2004). Growth patterns in young adult monozygotic twin pairs discordant and concordant for obesity.. Twin Res.

[13] Wells PS, Anderson JL, Scarvelis DK, Doucette SP, Gagnon F (2006). Factor XIII Val34Leu variant is protective against venous thromboembolism: a HuGE review and meta-analysis.. Am J Epidemiol.

[14] Okada T, Nishizawa H, Kurata A, Tamba S, Sonoda M (2008). URB is abundantly expressed in adipose tissue and dysregulated in obesity.. Biochem Biophys Res Commun.

[15] Ichinose A, Davie EW (1988). Characterization of the gene for the a subunit of human factor XIII (plasma transglutaminase), a blood coagulation factor.. Proc Natl Acad Sci U S A.

[16] Muszbek L, Bagoly Z, Bereczky Z, Katona E (2008). The involvement of blood coagulation factor XIII in fibrinolysis and thrombosis.. Cardiovasc Hematol Agents Med Chem.

[17] Greco D, Kotronen A, Westerbacka J, Puig O, Arkkila P (2008). Gene expression in human NAFLD.. Am J Physiol Gastrointest Liver Physiol.

[18] Cheung VG, Conlin LK, Weber TM, Arcaro M, Jen KY (2003). Natural variation in human gene expression assessed in lymphoblastoid cells.. Nat Genet.

[19] Dahlman I, Linder K, Arvidsson Nordstrom E, Andersson I, Liden J (2005). Changes in adipose tissue gene expression with energy-restricted diets in obese women.. Am J Clin Nutr.

[20] Bujalska IJ, Quinkler M, Tomlinson JW, Montague CT, Smith DM (2006). Expression profiling of 11beta-hydroxysteroid dehydrogenase type-1 and glucocorticoid-target genes in subcutaneous and omental human preadipocytes.. J Mol Endocrinol.

[21] Recinos A, Carr BK, Bartos DB, Boldogh I, Carmical JR (2004). Liver gene expression associated with diet and lesion development in atherosclerosis-prone mice: induction of components of alternative complement pathway.. Physiol Genomics.

[22] Manevich Y, Reddy KS, Shuvaeva T, Feinstein SI, Fisher AB (2007). Structure and phospholipase function of peroxiredoxin 6: identification of the catalytic triad and its role in phospholipid substrate binding.. J Lipid Res.

[23] Ciullo M, Nutile T, Dalmasso C, Sorice R, Bellenguez C (2008). Identification and replication of a novel obesity locus on chromosome 1q24 in isolated populations of Cilento.. Diabetes.

[24] Kolehmainen M, Salopuro T, Schwab US, Kekalainen J, Kallio P (2008). Weight reduction modulates expression of genes involved in extracellular matrix and cell death: the GENOBIN study.. Int J Obes (Lond).

[25] Kimura R, Ohashi J, Matsumura Y, Nakazawa M, Inaoka T (2008). Gene flow and natural selection in oceanic human populations inferred from genome-wide SNP typing.. Mol Biol Evol.

[26] Clark JT, Kalra PS, Crowley WR, Kalra SP (1984). Neuropeptide Y and human pancreatic polypeptide stimulate feeding behavior in rats.. Endocrinology.

[27] Aoki K, Sun YJ, Aoki S, Wada K, Wada E (2002). Cloning, expression, and mapping of a gene that is upregulated in adipose tissue of mice deficient in bombesin receptor subtype-3.. Biochem Biophys Res Commun.

[28] Kaprio J, Pulkkinen L, Rose RJ (2002). Genetic and environmental factors in health-related behaviors: studies on Finnish twins and twin families.. Twin Res.

[29] Pajukanta P, Lilja HE, Sinsheimer JS, Cantor RM, Lusis AJ (2004). Familial combined hyperlipidemia is associated with upstream transcription factor 1 (USF1).. Nat Genet.

[30] Soro A, Pajukanta P, Lilja HE, Ylitalo K, Hiekkalinna T (2002). Genome scans provide evidence for low-HDL-C loci on chromosomes 8q23, 16q24.1-24.2, and 20q13.11 in Finnish families.. Am J Hum Genet.

[31] Aromaa A, Koskinen S, Kansanterveyslaitos (2004). Health and functional capacity in Finland: baseline results of the Health 2000 health examination survey..

[32] Zimmet P, KG MMA, Serrano Rios M (2005). [A new international diabetes federation worldwide definition of the metabolic syndrome: the rationale and the results].. Rev Esp Cardiol.

[33] Purcell S, Neale B, Todd-Brown K, Thomas L, Ferreira MA (2007). PLINK: a tool set for whole-genome association and population-based linkage analyses.. Am J Hum Genet.

