# Supplementary figures and images for: Use of Genome-Wide Expression Data to Mine the “Gray Zone” of GWA Studies Leads to Novel Candidate Obesity Genes
Source: PLoS Genet. 2010 Jun 3;6(6):e1000976. doi: 10.1371/journal.pgen.1000976 (PMC2880558; doi:10.1371/journal.pgen.1000976)

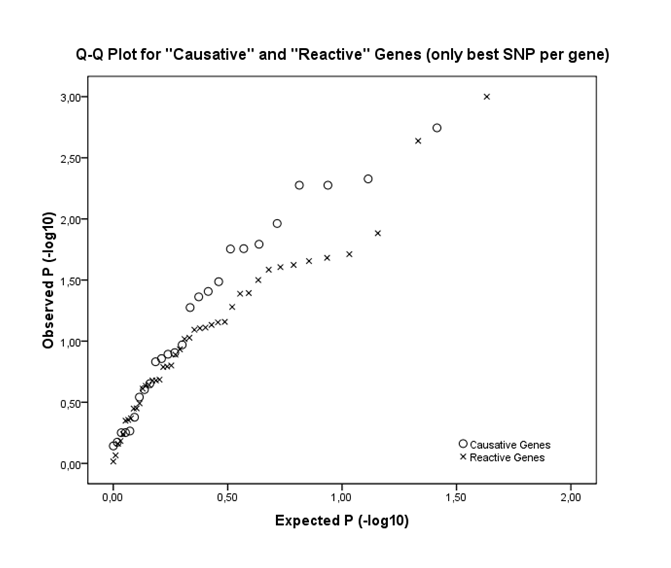

Supplement: Figure S1 — Q-Q plot for causative and reactive genes (only best SNP per gene). (0.06 MB TIF) [file pgen.1000976.s001.tif]

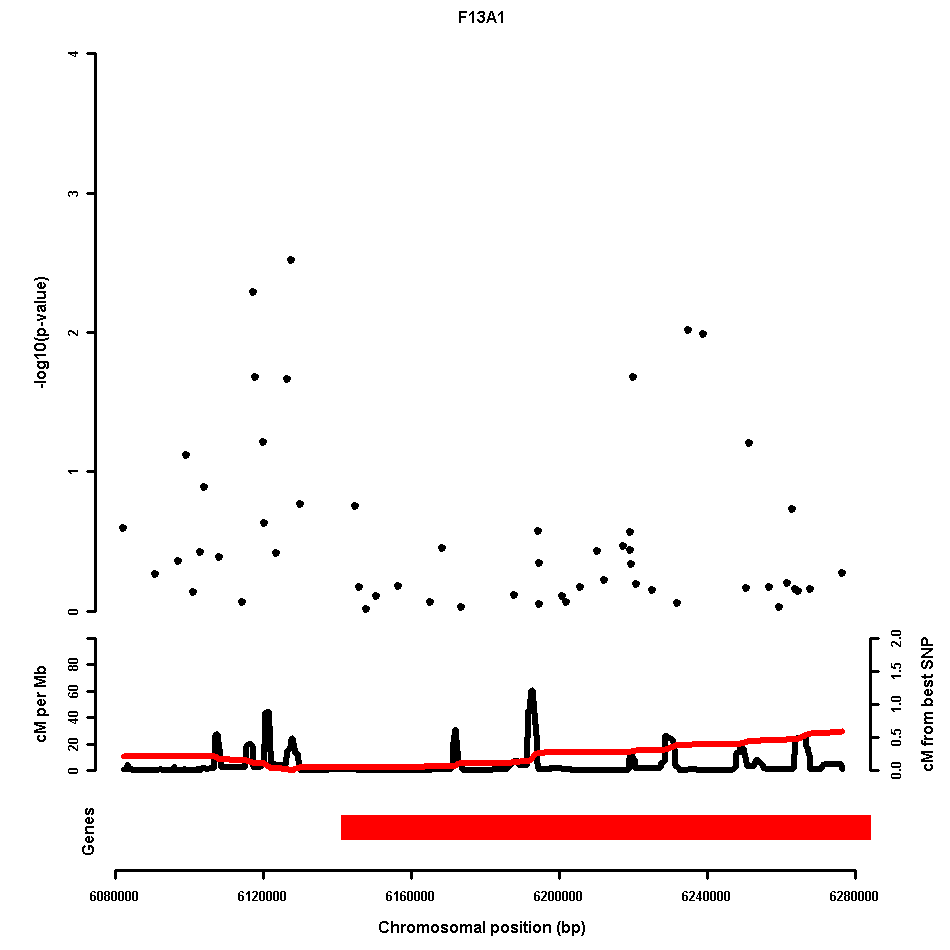

Supplement: Figure S2 — Association signals in F13A1. The upper panel displays the genotyped SNPs and their −log10 p-values. The panel below this displays the LD structure with a black line indicating the cM/Mb rate. High peaks correspond to recombination hotspots (with low LD). The red line indicates the genetic distance from the SNP with the lowest p-value. In the bottom most panel the red bar marks the location of the F13A1 gene. (2.71 MB TIF) [file pgen.1000976.s002.tif]

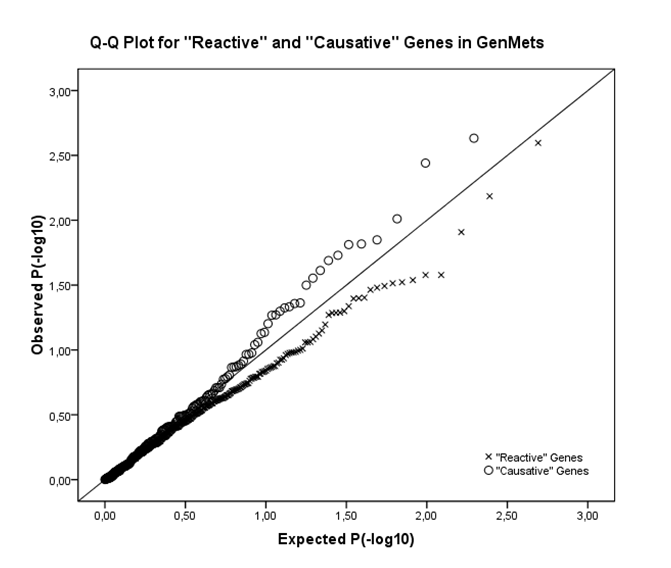

Supplement: Figure S3 — Q-Q plot of reactive and causative genes in the GenMets study. (0.07 MB TIF) [file pgen.1000976.s003.tif]
