# Supplementary material for: Use of Genome-Wide Expression Data to Mine the “Gray Zone” of GWA Studies Leads to Novel Candidate Obesity Genes
Source: PLoS Genet. 2010 Jun 3;6(6):e1000976. doi: 10.1371/journal.pgen.1000976 (PMC2880558; doi:10.1371/journal.pgen.1000976)
Supplement: Table S1 — Physical and biochemical characteristics of the obesity-discordant monozygotic twin pairs (n = 13). (0.04 MB DOC) [file pgen.1000976.s004.doc]

**Table S1.** Physical and biochemical characteristics of the obesity-discordant monozygotic twin pairs (n=13).

|  | **Obese co-twins** | **Non-obese co-twins** | **P value^a^** |
| --- | --- | --- | --- |
| Birth weight (g)^b^ | 2715 (2368, 2930) | 2540 (2105, 2860) | 0.041 |
| Birth length (cm)^b^ | 48 (45, 48) | 47 (45, 49) | 0.25 |
| BMI at birth (kg/m^2^)^b^ | 12.0 (11.1, 13.1) | 11.3 (9.8, 12.5) | 0.12 |
| BMI at 16 y (kg/m^2^)^c^ | 21.7 (21.2, 22.3) | 22.0 (21.1, 22.6) | 0.15 |
| BMI at 18 y (kg/m^2^)^c^ | 23.8 (22.8, 24.9) | 23.6 (21.8, 25.0) | 0.29 |
| BMI (kg/m^2^) | 30.6 (29.2, 32.0) | 25.5 (24.8, 26.0) | 0.0015 |
| Percent body fat | 38.0 (32.6, 42.9) | 28.2 (23.3, 35.9) | 0.0015 |
| Subcutaneous fat (cm^3^) | 5113 (4376, 5821) | 2728 (2430, 3554) | 0.0015 |
| Intra-abdominal fat (cm^3^) | 993 (827, 1065) | 516 (380, 587) | 0.0015 |
| Liver fat (%) | 3.7 (2.0, 11.0) | 1.0 (1.0, 2.5) | 0.0016 |
| Fat cell diameter (μm) | 103.3 (95.5, 106.8) | 85.8 (82.5, 89.9) | 0.0022 |
| Serum insulin (mU/l) | 9 (6, 11) | 4 (3, 6) | 0.016 |
| M value (mg·kg fat free mass^-1^·min^-1^) | 5.5 (4.5, 6.9) | 8.8 (7.6, 10.4) | 0.0047 |

Data are median (interquartile range).

^a^ Obese vs. non-obese co-twins, paired Wilcoxon’s test.

^b^ n=12 pairs, ^c^ n=11 pairs
