# Supplementary material for: Use of Genome-Wide Expression Data to Mine the “Gray Zone” of GWA Studies Leads to Novel Candidate Obesity Genes
Source: PLoS Genet. 2010 Jun 3;6(6):e1000976. doi: 10.1371/journal.pgen.1000976 (PMC2880558; doi:10.1371/journal.pgen.1000976)
Supplement: Table S2 — SNPs associating (at P<0.05) with BMI among the putatively causative genes in the GenMets study, ordered by overall P-value. (0.04 MB DOC) [file pgen.1000976.s005.doc]

**Table S2. SNPs associating (at *P*<0.05) with BMI among the putatively causative genes in the GenMets study, ordered by overall *P*-value.**

| **Gene name** | **MAF** | **SNP** | **Overall *P*-value** |
| --- | --- | --- | --- |
| *F13A1* | 0.341 | rs714408 | 0.0023 |
| *RORB* | 0.216 | rs11144062 | 0.0036 |
| *TSGA10IP* | 0.405 | rs491973 | 0.0098 |
| *HADHA* | 0.270 | rs1122972 | 0.0142 |
| *HADHA* | 0.271 | rs4665315 | 0.0152 |
| *HADHA* | 0.271 | rs962217 | 0.0154 |
| *F13A1* | 0.141 | rs3024358 | 0.0187 |
| *F13A1* | 0.192 | rs3024342 | 0.0205 |
| *F13A1* | 0.215 | rs5985 | 0.0244 |
| *HADHA* | 0.332 | rs10177371 | 0.0280 |
| *F13A1* | 0.213 | rs3116567 | 0.0317 |
| *TSGA10IP* | 0.426 | rs593525 | 0.0434 |
| *F13A1* | 0.280 | rs3024321 | 0.0439 |
| *HADHB* | 0.335 | rs11685458 | 0.0464 |
| *F13A1* | 0.183 | rs3024443 | 0.0474 |

Table has been sorted according to the overall *P*-value

MAF: minor allele frequency
